# Host-directed therapy against mycobacterium tuberculosis infections with diabetes mellitus

**DOI:** 10.3389/fimmu.2023.1305325

**Published:** 2024-01-08

**Authors:** Li Zhao, Ke Fan, Xuezhi Sun, Wei Li, Fenfen Qin, Liwen Shi, Feng Gao, Chunlan Zheng

**Affiliations:** ^1^ Department of Tuberculosis III, Wuhan Pulmonary Hospital, Wuhan, Hubei, China; ^2^ Department of Endocrinology, Union Hospital, Tongji Medical College, Huazhong University of Science and Technology, Wuhan, Hubei, China

**Keywords:** mycobacterium tuberculosis, diabetes mellitus, host-directed therapy, drug-resistance, immune

## Abstract

Tuberculosis (TB) is caused by the bacterial pathogen *Mycobacterium tuberculosis (MTB)* and is one of the principal reasons for mortality and morbidity worldwide. Currently, recommended anti-tuberculosis drugs include isoniazid, rifampicin, ethambutol, and pyrazinamide. TB treatment is lengthy and inflicted with severe side-effects, including reduced patient compliance with treatment and promotion of drug-resistant strains. TB is also prone to other concomitant diseases such as diabetes and HIV. These drug-resistant and complex co-morbid characteristics increase the complexity of treating MTB. Host-directed therapy (HDT), which effectively eliminates MTB and minimizes inflammatory tissue damage, primarily by targeting the immune system, is currently an attractive complementary approach. The drugs used for HDT are repositioned drugs in actual clinical practice with relative safety and efficacy assurance. HDT is a potentially effective therapeutic intervention for the treatment of MTB and diabetic MTB, and can compensate for the shortcomings of current TB therapies, including the reduction of drug resistance and modulation of immune response. Here, we summarize the state-of-the-art roles and mechanisms of HDT in immune modulation and treatment of MTB, with a special focus on the role of HDT in diabetic MTB, to emphasize the potential of HDT in controlling MTB infection.

## 1 Introduction

Tuberculosis (TB), caused by infection with Mycobacterium tuberculosis (MTB), remains one of the major health problems worldwide, with millions of new cases and more than one million deaths from TB each year (1). The combination of Bacillus Calmette-Guerin (BCG) vaccination and multiple antibiotics is clinically used as a strategy to control the transmission and progression of TB. However, BCG vaccination usually fails to achieve the desired protective effect, and treatment of TB with drug combinations such as isoniazid, rifampicin, pyrazinamide, and ethambutol requires up to 6-9 months of standard treatment (2). TB treatment is lengthy and has serious side effects, including reduced patient compliance with treatment and promotion of drug-resistant strains. Treating drug-resistant strains of TB is much more difficult compared to common infections (3). Alarmingly, drug resistance to TB is becoming a knotty issue.

At the same time, TB is also prone to other concomitant diseases such as diabetes and HIV (4, 5). Epidemiologic studies have shown that as many as 25-54% of newly diagnosed TB patients also have diabetes mellitus (DM) (6, 7), and up to 80% of patients with DM reside in low and middle-income level countries, which highly overlaps with the prevalence of TB (8). These drug-resistant and complex co-morbid characteristics increase the complexity of treating TB. Therefore, there is a need to develop and apply new therapeutic approaches to TB.

Host-directed therapy (HDT) is a novel adjunctive therapeutic strategy that has emerged in recent years in the treatment of TB infections. Unlike traditional antimicrobial therapy, HDT usually acts on the immune system of the host rather than the pathogens (9). Through specific modulation of the host immune system and immune processes, HDT can achieve the goal of limiting or enhancing the inflammatory response and interfering with host defense mechanisms utilized by pathogens. These immune effects enhance the killing effect on pathogens while attenuating pathological damage to the host, which is important for combating MTB infections, especially against drug-resistant MTB (10, 11). HDT reduces lung inflammation, improves focal drug penetration, and induces antimicrobial activity of phagocytes, thereby protecting the lungs, improving long-term survival, and shortening treatment time.

Consequently, HDT helps to address the current problems of complicated TB treatment procedures and the increase of drug-resistant strains (12). In this review, we comprehensively characterize the immune features of MTB infection and focus on elucidating the mechanisms and drug candidates associated with HDT. Notably, we focus on the current state of studies on HDT in DM with MTB.

## 2 The landscape of immune responses in MTB infection

The first contact of MTB with the human body is called primary infection. Pulmonary tuberculosis is the most common consequence of MTB infection. In aerosolized form, MTB passes through the respiratory tract into the lungs and in most cases enters the distal alveolar cavities (13). MTB can infect respiratory epithelial cells or mucosal tissues and reach the alveoli with two main fates: phagocytosis by alveolar-resident immune cells such as macrophages/dendritic cells (DCs) or successful infection of alveolar epithelial cells. The latter may lead to further spread of MTB, triggering TB in other parts of the body (14, 15). In most primary infections, the immune system can either completely clear the invading MTB or control the infection by forming stable granulomas (16).

TB-specific granulomas are formed by the organized aggregation of different types of immune cells at the site of infection, in which there is a complex and dynamic interaction between MTB and the host (17). From the perspective of the host, granuloma formation effectively limits the proliferation of MTB and the progression of the disease, which is the outcome in most major infected individuals. As for MTB, granulomas, while limiting the proliferation and spread of MTB within the host, allow MTB to remain dormant without being completely cleared by the host immune system, creating a state of latent TB infection reserving the possibility of subsequent reactivation (18–20).

When the immune function of the host is impaired, the core of the granuloma undergoes caseous changes and liquefaction in response to the accumulation of foam cells transformed from macrophages and a large number of necrotic host immune cells (21, 22). This change markedly diminishes the ability of the granuloma to restrict MTB, which may lead to bacterial release and disease progression at any time (23).

### 2.1 Innate immunity in MTB infection

#### 2.1.1 Macrophages

Macrophages are an important component of TB pathogenesis, participating in early exposure to MTB and acting as a refuge for MTB during chronic infection. When MTB invades, alveolar macrophages rapidly recognize microbe-associated molecular patterns (MAMPs) on the surface of MTB through pattern recognition receptor (PRR), and engulf MTB into the phagosome for acidic clearance, while recruiting other types of macrophages and participating in antigen presentation (24–26). Macrophages can secrete a variety of cytokines and chemokines, such as tumor necrosis factor (TNF)-α, interleukin (IL)-1β, IL-6, IL-23, and granulocyte-macrophage colony-stimulating factor (GM-CSF), and also fight against MTB infection through apoptosis and autophagy (27). But, accordingly, MTBs can also resist their host clearance, such as by inducing the conversion of macrophages into foam cells or inducing macrophage polarization through the production of mycolic acid, for example. Strongly toxic MTB can also prevent the fusion and acidification of phagolysosomes, thus successfully converting macrophages into cells that assist them in evading the host immune response (28, 29). Several new studies have also found that MTB can exert its pathogenic ability by modulating host cell metabolism (30–33).

#### 2.1.2 Dendritic cells

DCs are involved in antigen processing and presentation of MTB and are key to bridging innate and adaptive immunity (34). With the help of DC-SIGN and toll-like receptor (TLR), DC accomplishes the recognition and self-activation of MTB (35). After activation, DC secretes IL-12 and upregulates the expression of MHC-I, MHC-II, CD 40, CD 54, CD58, and CD80, and then migrates to mediastinal lymph nodes, where it activates a Th1-type specific response marked by interferon (IFN)-γ secretion through antigen presentation (36–38). MTB can influence DC immune function by interacting with DCs and modulating the secretion of anti-inflammatory and pro-inflammatory factors (39, 40).

#### 2.1.3 Neutrophils

Neutrophils are the most abundant cell type in the respiratory secretions of patients with active TB and are the cells that can be rapidly recruited and infiltrated after MTB infection. Neutrophils appear to be a double-edged sword in MTB infection. Early in the infection, neutrophils are recruited toward the infected foci in response to various cytokines and chemokines and then exert anti-TB effects along with macrophages, by releasing non-targeted inflammatory mediators (41, 42). On the other hand, the greater number of neutrophils in the lungs correlates with increased pathological damage caused by TB (43). MTB not only induces necrosis of neutrophils, but these necrotic neutrophils phagocytosed by macrophages also promote intracellular survival of MTB (44).

#### 2.1.4 Natural killer cells

NK cells are a type of innate lymphoid cells with potent cytolytic capacity. NK cells can directly lyse infected cells through cytotoxicity or secrete cytokines to exert anti-infective effects (45). NK cells can be activated by macrophage stimulation and also secrete IFN-γ and IL-22, which act on macrophages to inhibit MTB intracellular growth by enhancing phagolysosomal fusion (46, 47). Not only that, but NK cells are also involved in TB granuloma formation. During MTB infection, the function of NK cells seems to be suppressed, but the exact mechanism of action remains to be further investigated (48, 49).

### 2.2 Acquired immunity in MTB infection

**Figure 1:**
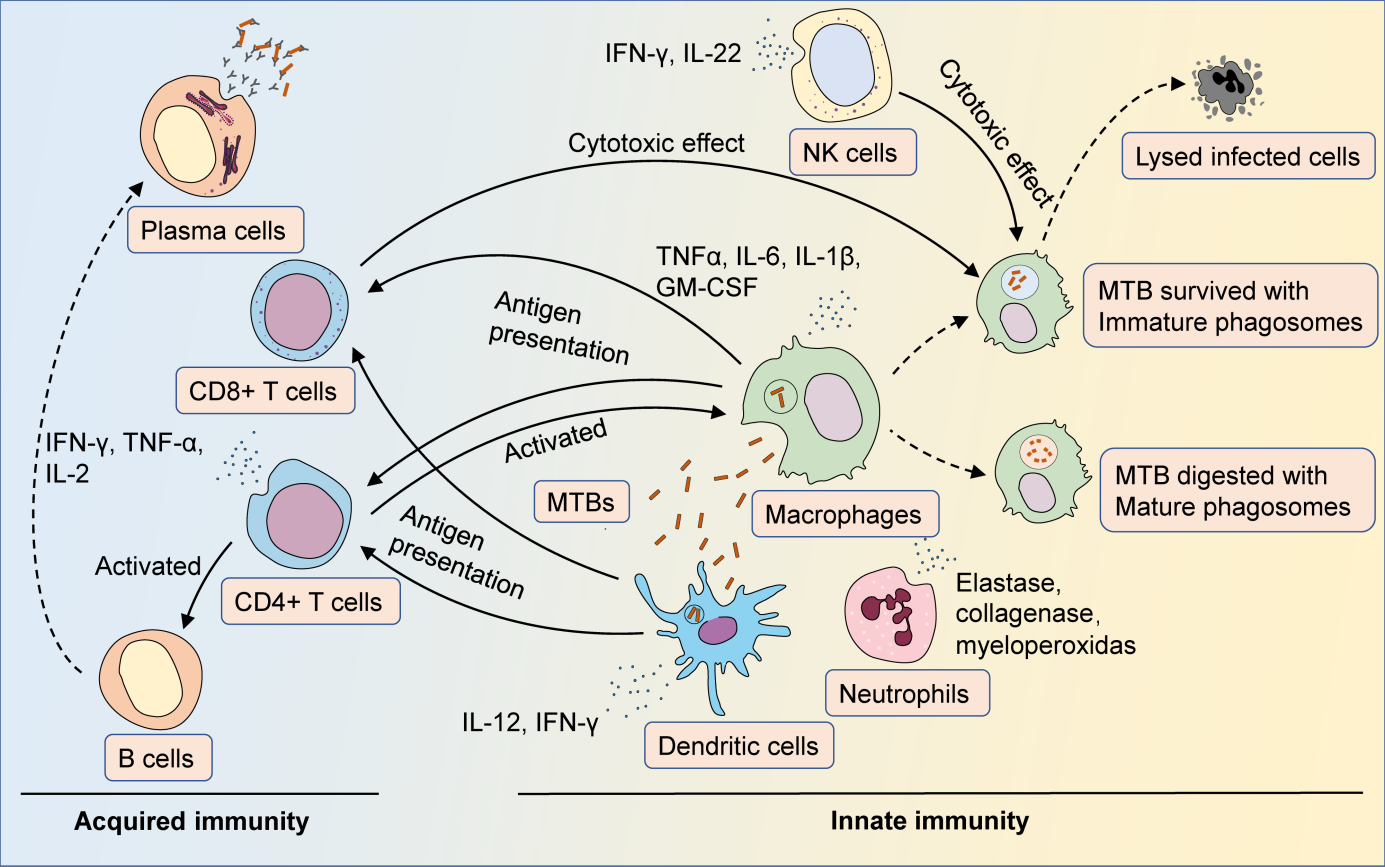
The immune response of MTB infection. After entering the body, MTB is first recognized by antigen-presenting cells such as macrophages and dendritic cells. Antigen-presenting cells phagocytose and process MTB, then activate T cells via antigen presentation, as well as secrete cytokines and chemokines to facilitate the initial immune response. Neutrophils could be recruited to the site of infection, fighting MTB infection by secreting cytokines and lysozyme. Macrophages are the major cells that fight MTB infection. Macrophages phagocytize MTB and eliminate bacteria by forming mature phagosomes. MTB could prevent itself from being eliminated by pathogen-host interactions that inhibit the maturation of phagosomes. Once activated, CD4+ T cells could trigger Th1- and Th17-type immune responses against MTB infection, and secrete cytokines such as IFN-γ, TNF-α, and IL-2 to enhance the antimicrobial activity of macrophages. Activated CD8+ T cells could synergize with NK/NKT cells to lyse infected cells through cytotoxic effects. Humoral immunity is also activated during MTB infection, with antibodies secreted by plasma cells assisting in countering the spread of the infection.

After the early response of innate immunity to MTB, adaptive immunity plays a key role in the control of MTB infection in the mid- and late stages. A variety of T cells, including CD4+ effector T cells, CD4+ regulatory T cells, and CD8+ cytotoxic T cells, are involved in the anti-MTB immune process (50). During MTB infection, macrophages and DCs recruited to the lungs could phagocytose and process MTB antigens and present them to CD4+ T cells after migration to secondary lymphoid organs. CD4+ T cells are stimulated by specific antigens caused by Tuberculosis (TB). Activated CD8+ T cells can secrete a variety of cytotoxic effector molecules together with NK/NKT cells to kill intracellular bacteria by lysing infected cells (51). Humoral immunity is protective against MTB infection (52). Specific neutralizing antibodies in the mucosal barrier alone could protect against MTB invasion, whereas the bacterial load in the lungs of the B-cell-depleted MTB-infected mouse model was significantly increased (53). Theoretically, every process from the invasion of MTB into the body to its eventual removal could be a potential target for HDT (**Figure 1**).

## 3 The candidates of HDT for MTB treatment

### 3.1 Novel targets of HDT and their mechanisms

Cytokines are a class of biologically active small molecular peptides or glycoproteins produced by cells that possess the ability to regulate intrinsic and adaptive immunity. The main cytokines involved in MTB infection, mainly include IFN-α, IFN-γ, IL-2, IL-6, IL-10, IL-13, IL-17, TNF-α, and chemokines (54). IL-17A regulated inflammatory factors and T-cell recruitment against MTB infection. In contrast, IL-27 suppressed IL-17 A production, thereby limiting the anti-TMB immune response. Blocking IL-27R exemplified its potential as an HDT (55). During acute MTB infection, IL-4i1 deficiency led to increased M1 polarization, nitrite and IFN-γ expression, which enhanced protection against infection and was expected to be an immunomodulatory target (56). Khan et al. found that activation of CD40 and TLR-4 induced increased secretion of IL-12, IL-6, and TNF-α and enhanced autophagy by DCs, as well as enhanced adaptive immunity and bactericidal effects of anti-TB drugs. These effects led to a significant reduction of bacterial load in the lungs (57). Notably, there is a correlation between plasma levels of eicosanoids and MTB infection status in patients with MTB (58). MTB infection elicited an imbalance of eicosanoids in the host, which in turn contributed to imbalanced IL-1 and IFN-γ expression, whereas IL-1 increased host resistance to MTB by inducing arachidonic acid-like and limiting excessive I-IFN production (59).

Histone deacetylase (HDAC), an important class of enzymes in histone modification, is involved in epigenetic regulatory processes (60). MTB promotes self-survival by affecting HDAC transcript levels, and inhibition of HDAC activity enhances macrophage MTB resistance (61). HDAC3 inhibitor RGFP966 could inhibit MTB growth through anti-microbial activity and modulation of macrophage pro-inflammatory factor secretion (62). SIRT1 and SIRT7 expression was down-regulated in MTB-infected macrophages. Cheng et al. found that SIRT1-induced phagocytosis of lysosomal-lysosomal fusion and autophagy was activated and inhibited the inflammatory response through RelA/p65 deacetylation, reducing the intracellular growth of MTB and MTB-resistant strains (63). Zhang et al. found that silencing SIRT7 expression could limit NO production in macrophages, reduce their frequency of early apoptosis, and promote intracellular growth of MTB, suggesting a candidate molecule for HDT.

Ferroptosis is a regulated necrosis triggered by a combination of iron toxicity, lipid peroxidation, and plasma membrane damage involved multiple infection-immunomodulatory processes (40, 64). Dai et al. found that MTB infection enhanced intracellular MTB iron utilization and promoted its proliferation through TRIM21- and NCOA4-dependent ferritinophagy. This confirmed the effectiveness of ferritin metabolism as an anti-MTB target (65). Amaral et al. found that infection of GPX4-deficient mice with MTB produced higher levels of lung necrosis and bacterial load. GPX4-deficient macrophages infected with MTB exhibited necrosis, suggesting the involvement of ferroptosis in MTB infection and the possibility of glutathione (GSH)/GPX4 as an HDT target for MTB (66).

In macrophages, galectin-8 recognized damaged MTB-containing phagosomes and then targeted MTBs for selective autophagy (67). Galectin-8 appeared to be required for targeting and controlling MTB in macrophages. Alarmin S100A8/A9 mediated neutrophil accumulation by upregulating CD11b expression during progression to chronic TB, leading to worse disease outcomes (68). SP110b is an IFN-induced nuclear protein that governs host innate immunity to MTB infection. SP110b regulated the activity of NF-κB to inhibit IFN-γ-mediated monocyte and macrophage death, effectively controlling the growth of MTB while reducing inflammatory lesions in the lungs (69). MTB promoted intracellular survival in macrophages by elevating triacylglycerol (TAG) storage in macrophages via the WNT6/ACC2 pathway and could be considered as an HDT target (70). Pires et al. demonstrated that silencing Cystatin C significantly increased intracellular killing of MTBs by macrophages, with a concomitant promotion of HLA-II expression and IFN-γ secretion, and CD4+ T cell proliferation in macrophages (71). The use of siRNA Cystatin F produced a similar effect (72). Smyth et al. found that MTB infection induced up-regulation of protein kinase R (PKR) expression and activation in macrophages and inhibited intracellular MTB survival through activation of IFN-γ-mediated autophagy, confirming the validity of PKR as an HDT target (73). Inhibition of transglutaminase 2 (TG2) could limit MTB growth within macrophages (74). TG2 inhibitors can inhibit MTB growth by affecting macrophage autophagy and inhibit granuloma growth (75). In addition, inhibition of PARP is a potential strategy to effectively alter macrophage chemokine secretion and inhibit MTB and multidrug-resistant (MDR)-MTB levels, which could be used to develop HDT therapies targeting intracellular MTBs (76).

### 3.2 NcRNA-related HDT

Non-coding RNAs (ncRNAs) account for 98% of total RNAs and are the major RNA transcripts in organisms. These transcribed sequences mainly include lncRNAs, miRNAs, and circRNAs, which generally do not encode proteins but play regulatory roles at the transcriptional or post-transcriptional level (77). NcRNAs interact with each other in a complex network of roles. Currently, specific ncRNA species and expression levels abnormally occur in MTB infection, and these ncRNAs may be involved in the regulation of host-MTB interactions, and thus are expected to be potential candidates for HDT.

MTB could promote its survival by affecting the activity of lysosomal tissue proteases in host macrophages (78). Notably, miR-106b-5p, specifically up-regulated by MTB, was involved in the process of MTB on lysosomal tissue protease activity, which was achieved by binding to CTSS mRNA. Knockdown of miR-106b-5p in infected cells significantly increased the expression and activity of CTSS, as well as the level of T-cell activation and intracellular killing of MTB by macrophages. Additionally, the expression levels of miR-143 and miR-365 were significantly increased in MTB-infected macrophages (79). Knockdown of the expression of both could reduce the production of IL-6 and CCL-5, inhibit the proliferation and growth of MTB in macrophages, and induce apoptosis in infected macrophages. Further, BACH-1 and C-Maf were direct targets of miR-143, whereas miR-365 directly targeted cMaf, Bach1, and Elmo-1. Fu et al. demonstrated that the miR-342-3p/SOCS6 axis could affect the expression of inflammatory cytokines and chemokines and regulate the transition between MTB-induced apoptosis and necrosis by mediating K48-linked ubiquitination and RIPK3 degradation via A20 (80). Overexpression of MiR-342-3p improved resistance to MTB in mice. MiR-27a was abundantly expressed in MTB-infected animal, human, and macrophage cells (81). MiR-27a inhibited Ca2+ signaling by targeting the ER-located Ca2+ transporter protein CACNA2D3. Down-regulation of miR-27a levels increased resistance to MTB infection in mice. Targeting these miRNAs may be able to serve as the candidates for HDT.

LncRNAs are a category of ncRNAs with lengths greater than 200 nt, characterized by tissue-specific and spatiotemporal specificity, and low sequence conservation (82). In MTB infection, the expression signature of lncRNAs associated with immunoregulatory pathways is dysregulated (83). Gcanga et al. revealed that the expression of lincRNA-MIR99AHG correlated with the polarization status of macrophages (84). Lower levels of lincRNA-MIR99AHG were present in macrophages infected with MTB and in peripheral blood monocyte-derived macrophages from patients with active TB, and its expression was induced by stimulation of macrophages with IL-4/13. Knockdown of lincRNA-MIR99AHG significantly reduced intracellular MTB growth and proinflammatory cytokine production in mouse/human macrophages and reduced MTB load in mouse lungs and spleens.

### 3.3 Potential HDT agents for MTB treatment

Ibrutinib is a classical anti-chronic lymphocytic leukemia agent. Hu et al. found that ibrutinib induced macrophage autophagy by inhibiting the BTK/Akt/mTOR pathway, inhibited intracellular MTB growth in macrophages, and significantly attenuated MTB loading in mice (85). Boland et al. proved that the antitumor agent tamoxifen, although it lacked a direct antimicrobial effect, could produce an anti-TB effect by inducing autophagy rather than targeting its classical estrogen receptor pathway (86). This finding was further validated in a zebrafish model of TB infection, confirming the HDT potential of tamoxifen.

Linezolid is used in anti-tuberculosis therapy but carries some host toxicity. Winchell et al. demonstrated that co-administration of IL-1 blockers and linezolid attenuated side effects resulting from linezolid treatment and enhanced the therapeutic effect of linezolid in MTB-infected rhesus monkeys (87). Pires et al. found that lysosomal proteases were down-regulated after MTB infection of macrophages, thereby promoting macrophage survival. Treatment with saquinavir induced an increase in lysosomal protease activity and promoted intracellular MTB killing, as well as improved the expression of HLA class II antigens and the secretion of IFN-γ, while promoting the activation and proliferation of T lymphocytes (88). They further found that liposomal saquinavir could improve the transformation efficiency and reduce the drug cytotoxicity, and significantly enhance the killing effect of macrophages against MDR and XDR strains (89). There have been no clinical trials of saquinavir as an HDT drug for the treatment of tuberculosis. However, Ribera et al. found that the combination of saquinavir, ritonavir, and rifampicin, may result in lower blood levels of saquinavir and ritonavir in patients (90). Considering the wide use of rifampicin in the treatment of TB, more studies are needed on the methodology and efficacy of the use of saquinavir as an HDT drug for TB.

The mammalian target of rapamycin (mTOR) is a serine/threonine protein kinase with a molecular weight of 289 kDa, which has been universally used for the treatment of cancer and against organ transplant rejection (91). By analyzing the gene expression of MTB-infected DCs, Etna et al. found that rapamycin could regulate the transcriptional expression of IFN and downstream products in a GSK-3β-dependent manner, as well as regulate the balance of pro- and anti-inflammatory factors (92). But another mTOR inhibitor everolimus showed unsatisfactory results. A relapse of tuberculosis was found in the renal transplant patient and the renal cell carcinoma patient treated with everolimus (93, 94). Additionally, the patient with a neuroendocrine pancreatic tumor developed two tuberculosis infections within a year and a half while being treated with everolimus (95). Studies of strains isolated from his body showed that although everolimus stimulated autophagy and the production of reactive oxygen species, it did not exhibit direct or indirect antimicrobial activity against MTB. Although everolimus promoted the recovery of lung function in a phase II clinical trial evaluating the effectiveness of everolimus as HDT in the treatment of TB (96), further preclinical and clinical studies are needed to determine the safety and scope of its use as HDT for TB.

**Figure 2:**
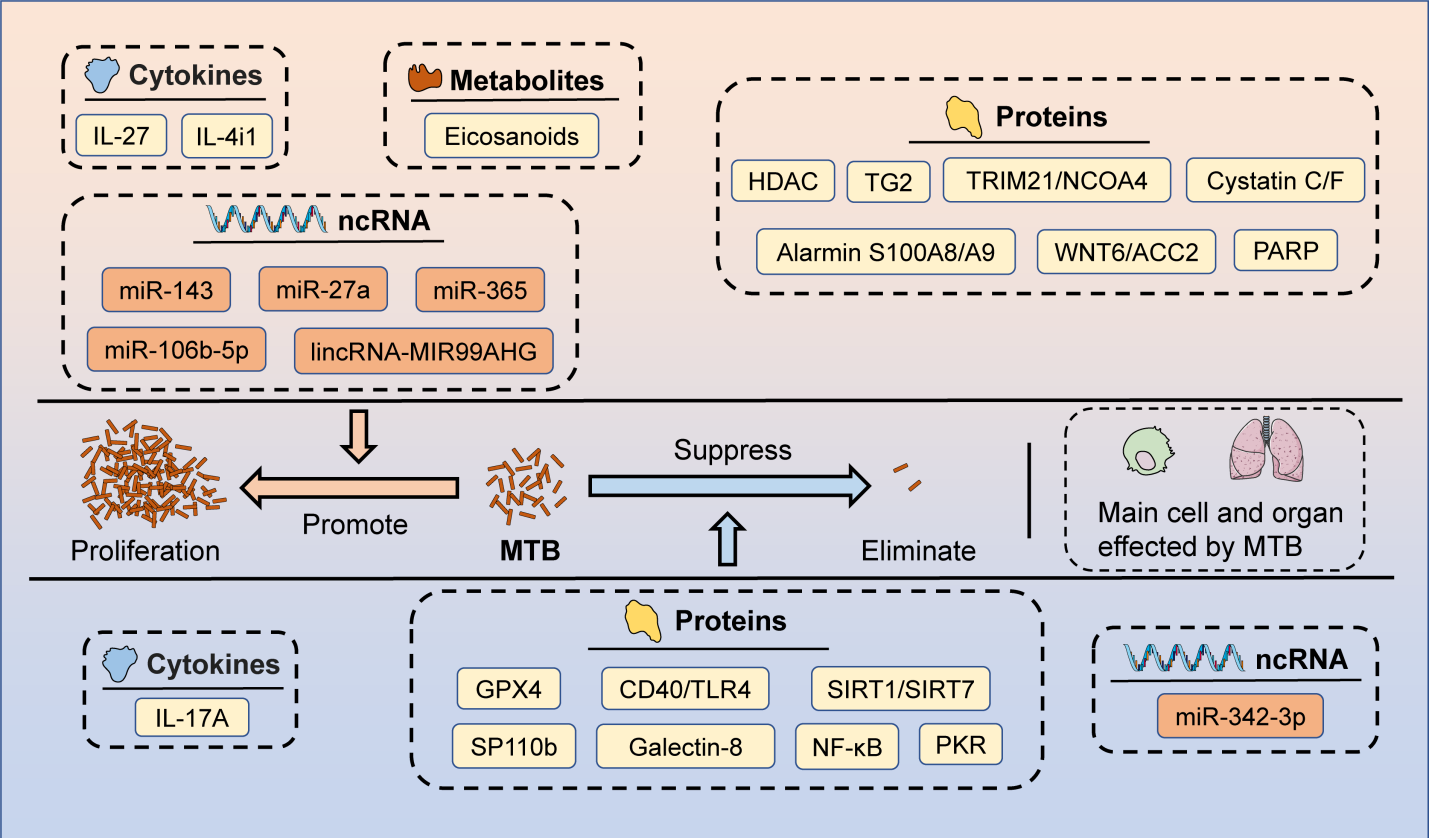
The potential targets of HDT for MTB infection. Macrophages and lungs are the primary cells and organs affected by MTB after the invasion of the body. Several candidate targets have been identified that could potentially be used for HDT, including cytokines (promote MTB proliferation: IL-27 and IL-4i1; inhibit MTB proliferation: IL-17A), metabolites (promote MTB proliferation: Eicosanoids), proteins (promote MTB proliferation: HDAC, TG2, TRIM21/NCOA4, Cystatin C/F, Alarmin S100A8/A9, WNT6/ACC2, and PARP; inhibit MTB proliferation: GPX4, CD40/TLR4, SIRT1/SIRT7, SP110b, Galectin-8, NF-κB, and PKR), and ncRNAs (promote MTB proliferation: miR-143, miR-27a, miR-365, miR-106b-5p, and lincRNA-MIR99AHG; inhibit MTB proliferation: miR-342-3p).

Appropriate levels of prostaglandin E2 (PGE2) are necessary to induce an optimal innate immune response early in MTB infection. Inhibition of cyclooxygenase-2 (COX-2) with celecoxib significantly reduced PGE2, enhanced IFN-γ production and NO release, and increased phagocytosis of MTB by macrophages (97). However, Mortensen et al. showed in different mouse models that although COX inhibitor treatment was commonly used to alleviate TB symptoms, it actually impaired the Th1-type immune response after MTB infection, reduced Th1 differentiation and IFN-γ expression, and decreased the protective capacity of CD4+ T cells against overt transfer (98). Mo et al. evaluated the effects of 4 calcium modulators on MTB in macrophages and found that flunarizine treatment could enhance macrophage bactericidal capacity by promoting maturation and acidification of phagolysosomes by increasing CaM and pCaMKII levels (99) (**Figure 2**).

### 3.4 HDT in combination with conventional drugs

Adjuvant HDT is expected to be a potentially innovative alternative to improve the efficacy of conventional antimicrobial therapy for MTB. Some immunomodulators or compounds can effectively improve the concentration and effect of therapeutic drugs such as rifampicin, functioning as sensitizers with therapeutic safety for MTB therapy. For example, Subbian et al. prepared a rabbit model infected with virulent MTB tuberculosis using aerosol exposure and administered a combination regimen of phosphodiesterase-4 inhibitor (PDE4i) CC-11050 and isoniazid (100). INH + CC-11050 treatment was more efficient than isoniazid alone in terms of increased MTB clearance efficiency, and the inflammatory state was suppressed, as evidenced by the downregulation of inflammatory factors such as TNF-α. Previously, they had demonstrated the effectiveness of another PDE4i, CC-3052, in combination with isoniazid for the treatment of rabbit tuberculosis (101, 102). Similarly, in a mouse model, they also confirmed the effectiveness of PDE4-i CC11050/3052 in controlling the inflammation in the lungs of MTB-infected mice (103, 104). These demonstrated that PDE4-i adjuvant therapy significantly improved the response to isoniazid treatment in model with experimental tuberculosis and could be a candidate for HDT. In a Phase II clinical trial, CC11050 was shown to have a favorable safety and tolerability profile in the adjuvant treatment of tuberculosis, along with facilitating the recovery of lung function (96). Enhancement of NOD-2 and TLR-4 signaling contributed to the enhancement of host immunity in a mouse model of MTB, and reduced the dose and duration of rifampicin and isoniazid treatment (105).

### 3.5 Newly discovered natural compounds of HDT

Natural products are substances naturally produced by organisms that are pharmacologically or biologically active. Natural products have been used since ancient times to treat human diseases. Recent advances in modern science and technology have made it possible to purify and identify the active ingredients in natural products, thus further improving the efficiency of their utilization (106, 107). Several studies have focused on the utilization of active ingredients in natural products for HDT, and some progress has been made.

Andrographolide inhibited the Notch1/Akt/NF-κB signaling pathway, contributing to the inhibition of NLRP3 inflammasome activation and subsequent IL-1β production, which probably facilitated the improvement of macrophage resistance to MTB infection (108). Ursolic acid boosted macrophage autophagy and enhanced intracellular killing of MTB by synergistically inhibiting Akt/mTOR and TNF-α/TNFR1 (109). Therefore, ursolic acid may be a potential adjuvant drug for anti-tuberculosis HDT by virtue of its macrophage-inhibiting, inflammation-suppressing function. Baicalin was capable of inducing autophagy activation in MTB-infected macrophages by inhibiting the PI3K/Akt/mTOR pathway. This further limited the generation of inflammation-associated NLRP3 inflammasome and IL-1β. Thus, baicalin exhibited anti-inflammatory and anti-MTB functions with HDT potency (110). Baicalein promoted autophagy by inhibiting the Akt/mTOR pathway, downregulating AIM2 and NLRP3 inflammasomes, and inhibiting pyroptosis in MTB-infected macrophages (111). The immune function and antimicrobial capacity of baicalein made it a candidate for HDT in treating MTB.

The marine natural product Clionamines stimulated macrophage autophagy by down-regulating PI4KB and inhibited MTB survival in macrophages (112). Guttiferone K isolated from Yunnan Baiyao was able to inhibit the downstream NF-κB and Akt/mTOR signaling pathways via the TLR/IRAK-1 pathway, thereby significantly inducing autophagy and suppressing MTB-induced inflammatory responses in macrophages (113). Berbamine was shown to activate macrophage autophagy by modulating the ROS/Ca2+ axis, and could efficiently inhibit MTB, demonstrating the advantage of improving the efficacy or shortening the course of treatment in drug-resistant TB (114). The antigout drug colchicine enhanced macrophage resistance to MTB by modulating NLRP3-dependent IL-1β signaling and COX-2-mediated PGE2 production in macrophages (115).

Negi et al. investigated that the β-glucan polysaccharide, curdlan, activated STAT-1 to induce inducible nitric oxide synthase (iNOS) and NO expression, thereby enhancing the anti-MTB activity of macrophages (116). In a mouse model infected with MTB, curdlan activated protective Th1 and Th17 immunity and significantly reduced MTB load in the spleen and lung of mice. Polysaccharide-rich extract (PRE) isolated from Tinospora cordifolia treatment upregulated pro-inflammatory factor expression and activation of p38, ERK, JNK, and MAPK, and inhibited the survival of drug-resistant MTB in macrophages by inducing NO expression (117).

Curcumin, a component of traditional medicine derived from turmeric, is a lipophilic polyphenol. Curcumin has demonstrated impressive therapeutic efficacy in inflammatory, tumor, and infectious diseases, accompanied by low cost and excellent tolerability (118). Curcumin treatment induced autophagy and apoptosis in infected/uninfected MTB macrophages, with the conversion of LC3-I to LC3-II, the degradation of p62, and upregulation of the pro-apoptotic proteins Bax, Casp3, and PARP, as well as the reduced expression of the anti-apoptotic protein Bcl (119). CMN encapsulation in PLGA to form nanoparticles (ISCurNP) improved CMN bioavailability and more effectively assisted in killing MTBs in RAW 264.7 macrophages.

### 3.6 Nebulized drug delivery of HDT

Nebulized inhalation is a direct drug delivery method using the respiratory tract and lungs as target organs, with the advantages of rapid onset of action, high local concentration, easy application, and fewer systemic adverse effects (120). The use of inhaled drugs for MTB treatment is a potentially efficient therapeutic tool that is gradually receiving attention. For instance, the utilization of STAT3 inhibitor ST3-H2A2 or IL-10Ra inhibitor IL10R1-7, when administered in aerosol form, effectively reduced MTB load in mouse lungs and was accompanied by an increase in the anti-bactericidal activities of NOS, NADPH oxidase and lysozyme (121). Targeting the host lung IL-10-STAT3 pathway in aerosol form, potentiated host bactericidal capacity and modulated apoptosis and autophagy pathways, serving as a novel HDT option.

All-trans retinoic acid (ATRA), the most common metabolite of vitamin A, promotes transcriptional activation of differentiation-associated genes and plays an important role in the treatment of cancer, dermatological disorders, acute promyelocytic leukemia (APL), and MTB infections (122). In the treatment of MTB, ATRA can modulate the immune robustness of the host body by regulating the expression of specific genes, monocyte activation, and induction of autophagy, which are promising candidates for HDT. The application of ATRA may result in varying degrees of side-effects due to off-target effects, and therefore the development of different ATRA-loaded drug delivery systems is highly prospective. O’Connor et al. developed an ATRA-loaded microparticle (ATRA-MP) that could be inhaled via the respiratory tract and was better targeted for delivery to alveolar macrophages than ATRA solutions (123). In the MTB-infected mouse model, three doses of ATRA-MP administered intratracheally resulted in a significant reduction in lung MTB load and improved lung pathology. Furthermore, Bahlool et al. also constructed ATRA-loaded PLGA nanoparticles (ATRA-PLGA NPs) in HDT against Tuberculosis (124). ATRA-PLGA NPs could target and be delivered to THP-1 cells in large quantities, leading to inhibition of H37Ra MTB proliferation in macrophages in a dose-dependent manner. ATRA-PLGA NPs also reached high concentrations locally in the lungs and were ultimately uptaken and therapeutically acted upon by MTB-infected cells. These studies highlighted the targeting, efficacy, and safety of inhalable, controlled-release ATRA formulations in HDT, providing a novel therapeutic tool for MTB. Singh et al. designed liposomes targeting CD44 using thioaptamers (CD44TA-LIP) that modulated antigen presentation and inflammatory factor expression in macrophages, enhancing the ability to enhance the killing effect on MTB (125) (**Table 1**).

**Table 1:** Potential HDT drugs for TB therapy.

Type of mechanism	Drugs/compounds inhibit MTB	Mechanism	Effects	Phase of clinical research	Ref.
Modulate innate immunity/acquired immunity	NOD-2/TLR-4 activator	Enhanced host immunity, used with rifampicin or isoniazid	Reduced the dose and duration of rifampicin and isoniazid treatment	Under cellular/*in vivo* investigation	(105)
Modulate innate immunity/acquired immunity	Curdlan	Activated STAT-1 to induce iNOS and NO expression	Activated protective Th1 and Th17 immunity	Under cellular/*in vivo* investigation	(116)
Modulate innate immunity/acquired immunity	Metformin	Enhanced host cell activity and promoted inflammatory killing, used with isoniazid	Modulated cytokines and chemokines, curbed the growth of MTB	Clinical used drug, under cellular/*in vivo*/cohort investigation	(126–131)
Modulate Inflammation	PDE4i CC-11050/3052	Inhibited PDE4, used with isoniazid	Increased MTB clearance efficiency and controlled the inflammation in the lungs	Under phase II clinical trial	(96, 100–104)
Modulate Inflammation	Andrographolide	Inhibited Notch1/Akt/NF-κB pathway	Improved macrophage resistance to MTB infection	Under cellular investigation	(108)
Modulate Inflammation	Colchicine	Inhibited of IL-1β signaling and PGE2 production	Enhanced macrophage resistance to MTB	Under cellular/*in vivo* investigation	(115)
Modulate Inflammation	PRE	Upregulated pro-inflammatory factor expression and activation of p38, ERK and JNK MAPK, induced NO expression	Inhibited the survival of drug-resistant MTB in macrophages	Under cellular investigation	(117)
Modulate autophagy	Clionamines	Down-regulated PI4KB	Stimulated macrophage autophagy	Under cellular investigation	(112)
Modulate autophagy	Berbamine	Modulated the ROS/Ca2+ axis	Activated macrophage autophagy	Under cellular investigation	(114)
Modulate autophagy	Curcumin/ISCurNP	Induced the conversion of LC3-I to LC3-II, the degradation of p62 and the upregulation of the Bax, Casp3, and PARP, reduced the expression of the Bcl	Induced autophagy and apoptosis in infected/uninfected MTB macrophages	Under cellular investigation	(119)
Modulate inflammation and autophagy	Ursolic acid	Inhibited Akt/mTOR and TNF-α/TNFR1 pathway	Boosted macrophage autophagy and enhanced intracellular killing of MTB	Under cellular investigation	(109)
Modulate inflammation and autophagy	Baicalin	Inhibited PI3K/Akt/mTOR pathway	Induced autophagy activation in MTB-infected macrophages, limited the generation of inflammation-associated NLRP3 inflammasome and IL-1β	Under cellular investigation	(110)
Modulate inflammation and autophagy	Baicalein	Inhibited Akt/mTOR, AIM2 and NLRP3 inflammasomes	Promoted autophagy and inhibited the pyroptosis of macrophages infected with MTB	Under cellular investigation	(111)
Modulate inflammation and autophagy	Guttiferone K	Inhibited NF-κB and Akt/mTOR pathway	Suppressed MTB-induced inflammatory responses in macrophages	Under cellular investigation	(113)
Modulate inflammation and autophagy	ST3-H2A2 and IL10R1-7	Targeted IL-10-STAT3 pathway in aerosol form	Increased the anti-bactericidal activities of NOS, NADPH oxidase and lysozyme, reduced MTB load in mouse lungs	Under cellular/*in vivo* investigation	(121)
Modulate inflammation and autophagy	ATRA-MP	Targeted alveolar macrophages	Reduced lung MTB load and improved lung pathology	Under cellular/*in vivo* investigation	(123)
Modulate inflammation and autophagy	ATRA-PLGA NPs	Targeted THP-1 cells	Inhibited MTB proliferation in macrophages	Under cellular investigation	(124)
Modulate inflammation and autophagy	CD44TA-LIP	Modulated the function of macrophages	enhanced the ability to kill MTB	Under cellular/*in vivo* investigation	(125)

## 4 HDT for MTB treatment in diabetes mellitus condition

Diabetes is a complex metabolic disorder that impacts the functioning of several systems of the body. The link between DM and TB has been recognized for centuries (132). Several studies have confirmed that diabetic patients have a higher risk of contracting tuberculosis than non-diabetics, approximately two to eight fold (133). In 2021, about 400,000 people have already suffered from both tuberculosis and diabetes (134).

Type 1 diabetes mellitus is a typical autoimmune disease, in which specific activation of pancreatic β-cell-reactive T-cells is the key factor contributing to the pathologic changes. In the pathogenesis of type 2 diabetes, aberrant immune cell activation and the accompanying chronic inflammatory milieu are consequences of diabetes, which in turn lead to pathologic damage and disease progression. Thus, aberrant immunity plays an important role in disease progression in both type 1 and type 2 diabetes.

The characteristic hyperglycemia of diabetes cause a variety of immune response dysfunctions. The abundance of DCs in diabetic patients is significantly reduced, and their activation is also impaired (135, 136). Macrophages in a hyperglycemic environment could lead to higher levels of IL-6 and lower levels of IL-1β, as well as dysfunctional activation of the NLRP3 inflammasome (137). The conversion of macrophages from a pro-inflammatory to an anti-inflammatory phenotype is also impeded, leading to abnormal chronic inflammation in various organs, such as skin tissue wounds and pancreatic islets, resulting in their tissue damage and dysfunction (138, 139). Similarly, there is abnormal activation of neutrophils in diabetic patients, which damages tissues and organs by yielding increased amounts of ROS and pro-inflammatory cytokines (140, 141). Chronic hyperglycemia also causes NK cell dysfunction (142). In adaptive immunity, hyperglycemia could reduce the number of lymphocytes in the germinal centers and glycosylate immunoglobulins, thereby affecting their normal functions (143, 144). Upon antigenic stimulation, adaptive immunity in diabetic patients produces lower levels of immune responses, including reduced cell activation and proliferation as well as reduced cytokines (145, 146). These innate and adaptive immune deficiencies make diabetics more susceptible to infection, while some microorganisms are more nutrient-rich in a hyperglycemic environment and are more prone to uncontrollable infections (147). Thus, TB-diabetes complications are detrimental to the treatment of TB (148). Therefore, higher rates of treatment failure and higher mortality can be observed in patients with TB-DM than in patients with TB alone (149, 150). Meanwhile, patients with TB-DM are also more likely to develop resistance to MTB and a greater risk of transmission (151, 152).

On the one hand, MTB infection also influences the progression of diabetes, and it is well established that MTB infection leads to impaired glucose tolerance in the host, which is a key component in the pathogenesis of diabetes (153). Impaired glucose tolerance can be normalized in the host after successful treatment, but even latently infected MTB increases the risk of future diabetes, possibly by affecting insulin resistance in the host (154–156). A systematic review also concluded that TB promoted the onset and progression of diabetes (156). So, the state of co-morbidity of MTB and DM negatively affects the disease progression and outcome of both, which reinforces the difficulty and importance of their treatment.

### 4.1 The immune feature of MTB with diabetes mellitus

Imbalanced glycemic control impairs immunity to MTB infection. In MTB-infected diabetic patients, MTB antigen-induced levels of IFN-γ, TNFα, and IL-6 mRNA, were significantly higher, leading to an increased inflammatory response and worsened disease progression at the site of MTB infection.

The high-glycemic environment directly impacts the process of antigen processing and presentation, which in turn affects the clearance of MTB (157). Furthermore, T2DM altered the basal phenotype of human macrophages and diminished their capacity to respond, antigen expression, internalise, cytokines (IL-6, IL-1β, IL-10, and IL-12), and chemokines (MCP-1, MIG, and RANTES) secretion, and controlled MTB (158). T2DM significantly increased mortality, bacterial load in organs, and inflammatory lesions in a mouse model of MTB infection (159). Sinha et al. found that glucose tolerance-impaired mice were associated with more significant pulmonary pathologic changes, lower TNF-α, IFN-γ, IFN-β, and IL-10 levels, and a tendency toward higher bacterial loads after MTB infection. IFN-γ levels were restored after restoration of glucose tolerance following a normal diet. Prediabetes increased tuberculosis severity, but high body fat with unimpaired glucose tolerance was protective (160).

LTBi is associated with an increased risk of developing diabetes (161, 162). At the patient level, Masood et al. found that peripheral blood mononuclear cells from diabetic patients with latent TB (LTBi) could elicit a dysfunctional immune response upon stimulation with PPD. The peripheral blood mononuclear cells secreted higher levels of IFN-γ, IL-6, IL-2, TNF-α, and GM-CSF, accompanied by reduced SOCS3 mRNA expression. This suggested that LTBi was strongly associated with exacerbating immune dysregulation in diabetic patients (163). Kathamuthu et al. found that in MAIT cells of diabetic patients with latent infection of MTB, the expression of Th1 and Th 17-type cytokines was increased, while the expression of cytotoxicity markers PFN, GZE B, and GNLSN was downregulated (164). In LTB PDM/DM combined, there was a decrease in γδ T cells expressing cytokines, cytotoxicity, and other immune markers as well as elevated levels of cytokines in supernatants (165).

There could be an interaction between MTB infection and the development of DM, and MTB can also promote the progression of diabetes. Oswal et al. found that adipose tissue/cells could modulate lung pathology and bacterial loads in MTB-infected mice (166). MTB infection could induce adipocyte dysfunction, leading to increased lipid accumulation in the lungs and altering cellular energy metabolism through inhibition of signaling pathways such as insulin, AMPK, and mTOR (167). Podell et al. demonstrated using a guinea pig model that MTB infection alone induced insulin resistance and chronic hyperglycemia and resulted in the accumulation of AGEs in tissues and serum (168).

### 4.2 Potential targets and drugs for HDT in MTB with diabetes mellitus

T2DM mice exhibit increased body weight, blood glucose levels, glucose tolerance, insulin resistance, and increased susceptibility to MTB infection. Cheekatla et al. found that CD11c+ was the major source of IL-6 in MTB-infected T2DM mice, while NK-CD11c+ cell interactions increased IL-6 production. Anti-IL-6 treatment and anti-NK1.1 treatment significantly increased the survival rate of MTB-infected DM mice (169). The level of IL-22 was significantly lower in MTB-T2DM mice than in MTB mice (170). ILC3 was an important source of IL-22, and recombinant IL-22 treatment or overtransfer of ILC3 significantly improved the survival of MTB-T2DM-infected mice. The IL-22 pathway may be a novel target for therapeutic intervention in T2DM patients with active tuberculosis.

The level of macrophages response to MTB is also altered in metabolically altered groups. Macrophages in chronic diabetic patients show lower MTB killing capacity. Rapamycin activates macrophages to enhance anti-MTB activity independent of tuberculosis status, but this effect is poor in diabetic patients (171). Vrieling et al. found that plasma oxidized low-density lipoprotein (oxLDL) levels were significantly elevated in diabetic patients. *In vitro*, oxLDL could affect macrophage control of MTB by inducing lysosomal and autophagic dysfunction (172). Butyrate affected a variety of cellular functions, and its levels were altered in diabetic individuals. Lachmandas et al. found that physiological doses of butyrate were able to inhibit MTB-induced immune response *in vitro*, and blocking IL-10 reversed the effect of butyrate (173).

Oxysterol receptor GPR183 expression is reduced in diabetic patients infected with MTB and correlates with disease severity (174). Further studies in mice infected with MTB in the lung showed that macrophage targeting and recruitment to the site of infection was associated with activation of the GPR183/oxysterol axis. The lack of GPR183 in diabetic patients may be one of the important factors contributing to the difficulty in controlling their infections (175). Wang et al. constructed a T2DM-pulmonary tuberculosis (PTB) mouse model by injecting streptozotocin, nicotinamide, and MTB, and the expression of pSTAT3 was increased in T2DM-associated PTB mice. This confirmed that pSTAT3 could promote NFAT5 expression by inhibiting miR-19b/1281 transcription, which led to exacerbated lung injury in MTB-infected T2DM mice (176). Zhao et al. demonstrated that SP1 could activate Akt by inhibiting PTEN transcription, which exacerbated MTB-infected in diabetic mice infected with MTB (177).

**Figure 3:**
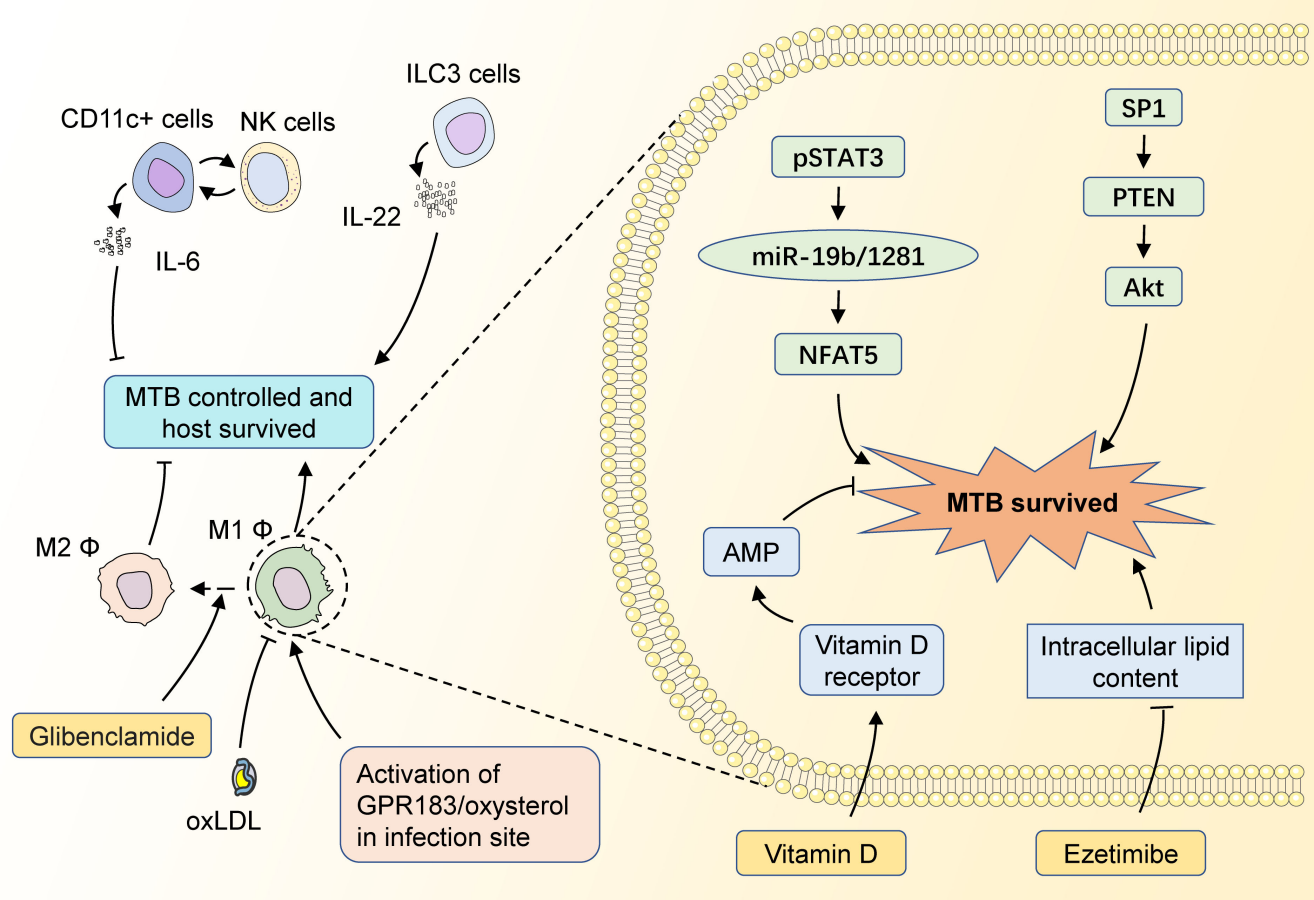
The potential targets of HDT for MTB infection with diabetic mellitus. CD11c+ cells could interact with NK cells to impair MTB control in the host by increasing the level of IL-6 secretion, while ILC3 cells could enhance the restriction of MTB infection by secreting IL-22. M1 macrophages could fight against MTB infection by secreting multiple inflammatory factors, and M2 macrophages have a significantly weakened ability to limit MTB. GPR183/oxysterol activated at the site of infection is necessary for macrophage recruitment, which facilitates the control of infection in the early stages. The diabetes-control drug Glibenclamide induces a shift from M1 to M2 macrophages, which adversely affects the control of MTB. OxLDL in the blood also impairs the antimicrobial capacity of M1 macrophages. pSTAT3 could promote NFAT5 expression by inhibiting miR-19b/1281 transcription, thus promoting the intracellular survival of MTBs. SP1 activates Akt by inhibiting PTEN transcription, which also impairs the MTB control of the host. MTB survival depends on the intracellular lipids, while Ezetimibe could inhibit MTB survival by decreasing the intracellular lipid contents. Meanwhile, Vitamin D could restrain MTB survival by inducing AMP expression.

Glibenclamide, a widely used sulfonylurea hypoglycemic agent, is an insulin secretion enhancer. Kewcharoenwong et al. found that pretreatment of monocytes with glibenclamide significantly decreased the levels of M1 surface markers (HLA-DR+ and CD 86+) and TNF-α production, and increased M2 (CD 163+ and CD 206+) surface markers and IL-10 expression. This resulted in impaired bactericidal capacity and potential susceptibility of primary human monocytes from T2DM patients to MTB (178). MTBs are able to invade host cells or survive within host cells by utilizing cholesterol, hence, some lipid-controlling drugs may have potential anti-TB effect (179, 180). Statins are one of the most widely used lipid-lowering drugs worldwide, which are extensively applied to reduce serum cholesterol levels and cardiovascular risk. Kang et al. investigated the effect of statins as adjunctive therapy for tuberculosis in a retrospective cohort study and found no significant association between statin use and the risk of developing tuberculosis in diabetic patients (181). Results from another cohort study by Lee et al. also showed that statin use was weakly associated with tuberculosis risk in diabetic patients > 65 years of age (182). However, in a study by Kim et al., it was found that statin users had a significantly lower risk of developing TB than non-statin users, although this protective effect would be attenuated by diabetes mellitus (183). This protective effect was confirmed by Pan et al., who also found that the higher the statin dose, the stronger the protective effect (184). The contradictions between the results of these large cohort studies may be due to some flaws in experimental design and data selection, and the presence of confounding factors may also be responsible. A series of preclinical studies and a number of clinical trials have demonstrated the potential therapeutic role of statins in non-diabetic tuberculosis patients (185–189). Accordingly, well-designed prospective studies are necessary to confirm the protective effect of statins in diabetic comorbidities with tuberculosis. Ezetimibe is an orally potent lipid-lowering drug that inhibits cholesterol absorption. Tsai et al. demonstrated that Ezetimibe reduced intracellular lipid content, and its treatment was associated with a reduced prevalence of latent TB and a significant inhibition of intracellular growth of MTB (190). Vitamin D can control MTB growth by inducing some anti-microbial peptides (AMP). Lopez-Lopez et al. found that vitamin D receptor (VDR) and AMP expression levels were lower in patients with T2DM, and supplementation with vitamin D was effective in improving their ability to clear MTB (191) (**Figure 3**).

It is worth noting that metformin is a first-line therapy for the management of diabetes mellitus type 2 (T2DM) with potent glucose-lowering properties, well-characterized biosafety, and relatively low cost (192). Metformin has been reported as an adjunctive therapy against MTB. Naicker et al. demonstrated that metformin modulated cellular interactions between MTB and macrophages by enhancing host cell activity and promoting inflammatory killing (126). The combination of metformin and isoniazid possessed a more potent ability to modulate cytokines and chemokines than isoniazid alone, effectively curbing the growth of MTB. Several clinical trials testing metformin as an adjunctive therapy for TB have been conducted. Numerous clinical studies have demonstrated that the use of metformin significantly reduced the incidence of active TB in diabetic patients, with a superior protective effect of high-dose metformin compared to low-dose metformin (127–129). Singhal et al. observed in two independent cohorts that MET-adjuvant therapy augmented the efficacy of conventional anti-TB drugs, thereby improving the severity of MTB infection and providing a better prognosis (130). Metformin reduced mortality in diabetic patients infected with tuberculosis during tuberculosis treatment, and the improved mortality rate was similar to that of non-diabetic tuberculosis patients (131).

GSH is an antioxidant, which is essential for the maintenance of redox homeostasis *in vivo*. It has been shown that GSH exhibits both direct antimycobacterial activity and in-direct antimycobacterial activity by enhancing the function of NK cells and T cells (47, 193, 194). In diabetic individuals, GSH levels are decreased (195). Liposomal glutathione (L-GSH) contains reduced GSH (rGSH) that could be readily absorbed and directly utilized by immune cells, resulting in rapid restoration of intracellular rGSH levels. Kachour et al. verified the therapeutic effects of L-GSH supplementation in a mouse model of tuberculosis, as manifested by a reduction in MTB burden and oxidative stress levels in the lungs, as well as an increase in lung levels of IL-12, IFN-γ, IL-2, IL-17, and TNF-α, and a decrease in IL-6, IL-10, and TGF-β production (196). Sasaninia et al. confirmed the favorable controlling effect of L-GSH supplementation in extrapulmonary tuberculosis in mice as well (197). (L-GSH supplementation has been shown to be an effective adjuvant therapy in both pulmonary TB and extrapulmonary TB). Beever et al. utilized a TB-diabetic mouse model to assess the therapeutic effects of combining L-GSH and rifampicin in TB (198). The results showed that L-GSH alone significantly reduced MTB burden and increased IFN-γ, TNF-α, IL-17, IL-10 and TGF-β1 levels. The combination of L-GSH and rifampicin provided better control of MTB infection and oxidative stress levels compared to rifampicin alone. In human-derived *in vitro* models, L-GSH also exhibited promising capacity in controlling MTB infection (195, 199). To et al. conducted a three month duration randomized controlled trial to evaluate the therapeutic efficacy of L-GSH in TB-DM, in which a total of 11 T2DM-positive subjects were enrolled in the L-GSH supplementation group and a total of 7 T2DM-positive subjects were enrolled in the placebo group (200). The use of L-GSH decreased the level of oxidative stress in the blood components of the subjects, with increased levels of Th 1-related cytokines IFN-γ, TNF-α, and IL-2, and decreased levels of IL-6 and IL-10. Despite the small sample size of this clinical trial, this results, as well as those of other clinical and preclinical trials, suggest that L-GSH supplementation has the potential to be an effective adjunctive therapy for the treatment of TB-DM (**Table 2**).

**Table 2:** Potential HDT drugs for TB-DM therapy.

Type of mechanism	Drugs/compounds inhibit MTB	Mechanism	Effects	Phase of clinical research	Ref.
Modulated innate immunity/acquired immunity	Liposomal glutathione	Maintained oxidative balance	Enhanced NK cell and T cell functions, regulated the secretion of inflammatory factors	Under phase II clinical trial	(196–200)
Modulated metabolism/innate immunity	Glibenclamide	Promoted macrophage M2 polarization	Impaired bactericidal capacity of macrophages and increased susceptibility to TB	Clinical used drug, under cellular investigation	(178)
Modulated metabolism/innate immunity	Statins	Modulated metabolism	Clear protective effect in non-diabetic TB patients, potential protective effect in diabetic TB patients	Clinical used drug, under phase IIA clinical trial	(181–189)
Modulated metabolism/innate immunity	Ezetimibe	Modulated metabolism	Decreased LTBI incidence and inhibited MTB intracellular survival	Clinical used drug, under cellular investigation	(190)
Modulated metabolism/innate immunity	Metformin	Modulated metabolism, used with isoniazid	enhanced host cell activity and modulated cytokine and chemokine secretion, enhanced the efficacy of anti-tuberculosis drugs, and decreased the incidence and mortality of TB	Clinical used drug, under cellular/*in vivo*/cohort investigation	(126–131)
Unknown	vitamin D	Unknown	Enhanced the ability of macrophages to eliminate MTB	Clinical used drug, under cellular investigation	(191)

## 5 Discussion

Systematically, this paper provides a meticulous elucidation of HDT treatment for MTB, including the immune mechanisms of MTB infection, potential therapeutic agents, and the immunological characteristics and potential HDT of diabetic MTB. Specifically, HDT can kill MTB by directly modulating immune response; second, HDT can modulate immune cells and factors in a multifaceted manner and does not involve pathogen-specific targets, limiting MTB to develop resistance and tolerance to HDT in the short term; third, HDT reduces the dosage and duration of antibiotics currently in use, thus improving patient adherence to treatment, and reduces the probability of drug-resistant strains. At present, HDT still holds several challenges that are worth attention.

First, there may be conflicting results between certain HDT treatment clinical trial data, possibly due to a variety of additional factors such as age, dose selection, subject lifestyle changes, race, co-infections, and genetic patterns. Therefore, more rigorously designed clinical trials are needed to validate the efficacy of HDT drugs. More importantly, it is difficult for mouse models to accurately simulate the immune response in humans infected with MTB. It is necessary to conduct a variety of other animal models of MTB infection to be able to validate the efficacy of HDT. These more accurate preclinical study data confirm the effectiveness of HDT and may provide a basis for clinical trials.

The activation of the immune system is a very finely tuned process. HDT involves the regulation of the immune system and needs to be applied with caution. Some immune targets have a narrow therapeutic window and require a high degree of accuracy in the dosage of the drug used. Enhancing the immune response may lead to aggravated histopathologic damage, while mitigating the immune response may lead to the spread and recurrence of infection. The development of novel HDT targets remains a necessity as there is still a lack of clinically available HDT drugs. The process of new target development needs to focus on the balance between destructive and protective immune responses, as well as the protection and reversal of organ tissues that have already produced pathological damage. Consideration has to be given to saving as much organ function as possible, the possibility of combining or avoiding interfering therapeutic effects with existing therapeutic modalities, as well as potential toxicity and safety issues. Especially in combination with other diseases, such as the MTB diabetic state, the organism may face a more complex immune environment. How to precisely regulate the immune response to achieve a balance between the therapeutic needs of different diseases is one of the difficult issues to overcome. Precise modulation of the immune response to achieve a balanced therapeutic need for different co-morbidities is the challenge to be overcome.

Finally, HDT may exhibit off-target effects. How to enhance the targeting ability of HDT drugs and avoid off-target effects on other biological processes in the body are areas of concern for future studies. This means that reliable biomarkers need to be developed for efficacy monitoring. Since individuals have different strengths of immune response to MTB infection and different cycles of treatment initiation, the selection of HDT type, dose, duration of use, mode of action, and combination drugs requires precise individualization, which is another issue that needs to be focused on in preclinical and clinical trials.

Overall, the drugs for HDT are repositioned drugs that have been approved and used in actual clinical practice, with a certain degree of relative safety and efficacy guaranteed. HDT is a potentially effective therapeutic tool for the treatment of MTB and diabetic MTB, and can compensate for the shortcomings of other TB therapies, including the reduction of drug resistance and modulation of immune response. In the context of co-morbidities, such as DM, HDT provides an individualized treatment tailored to individualized therapy. The application of HDT for the treatment of MTB and diabetic MTB warrants in-depth basic and clinical research.

## Author contributions

LZ: Investigation, Visualization, Writing – original draft. KF: Investigation, Visualization, Writing – original draft. XS: Writing – review & editing. WL: Writing – review & editing. FQ: Writing – review & editing. LS: Writing – review & editing. FG: Writing – review & editing. CZ: Writing – review & editing.
